# Metallothioneins in the Pathogenesis of Liver Diseases: A Review

**DOI:** 10.1155/ijh/8880889

**Published:** 2025-07-25

**Authors:** Anna A. Nowicka, Agnieszka Gomułkiewicz, Sylwia Serafińska, Krzysztof A. Simon, Piotr Dzięgiel

**Affiliations:** ^1^First Department of Infectious Diseases, J. Gromkowski Regional Specialist Hospital, Wroclaw, Poland; ^2^Clinical Department of Infectious Diseases and Hepatology, Wroclaw Medical University, Wroclaw, Poland; ^3^Division of Histology and Embryology, Department of Human Morphology and Embryology, Wroclaw Medical University, Wroclaw, Poland

**Keywords:** carcinoma, hepatocellular (HCC), cholestasis (PBC, PSC), fatty liver (MASLD), hepatitis, alcoholic (ArLD), hepatitis, autoimmune, hepatitis, viral, human, liver diseases, metallothioneins, oxidative stress, Wilson disease

## Abstract

Metallothioneins (MTs) are primarily known for maintaining cellular metal homeostasis and protecting against metal toxicity, but they also play critical roles in mitigating oxidative stress and modulating immune responses. Reduced MTs expression is associated with disease progression in several chronic hepatic disorders. In metabolic dysfunction–associated steatotic liver disease (MASLD), MT1 downregulation has been linked to the transition from simple steatosis to steatohepatitis. Additionally, evidence suggests that individuals with low MTs expression may be more susceptible to obesity, which is one of the key drivers in the development of MASLD. In chronic viral hepatitis, in vivo MTs downregulation contrasts with acute-phase in vitro upregulation, suggesting an effect of persistent inflammation. MTs downregulation in hepatocellular carcinoma (HCC) correlates with poor prognosis, positioning MTs as potential biomarkers and therapeutic targets. In contrast, increased MTs expression may play a protective or diagnostically informative role in other conditions. In alcoholic hepatitis, MT1 overexpression contributes to defense against oxidative stress and inflammation. In Wilson's disease, MTs demonstrates prominent hepatic expression and may offer diagnostic value alongside traditional markers such as rhodanine staining. In cholestatic liver diseases like PBC and PSC, MTs expression correlates with disease progression. This review highlights the emerging role of MTs in liver disease pathogenesis and positions them as promising molecular tools that may inform future strategies for diagnosis, prognosis, and treatment of liver diseases.

## 1. Introduction

Metallothioneins were initially discovered in 1957 through isolation from horse kidney tissue, with their role primarily described only as a cadmium (Cd) detoxifying agent [1]. Subsequently, MTs were identified as low-molecular-weight proteins exhibiting a notable affinity for other divalent metals, including zinc (Zn), copper (Cu), and mercury (Hg) [2].

In humans, four subfamilies of MTs are described: MT1, MT2, MT3, and MT4. MT1 and MT2, the most common isoforms, can be found in virtually all human organs, but in particular, high concentrations are present in the liver and kidneys. MT3 is primarily expressed in brain tissue and is also present in the reproductive organs, heart, retina, kidneys, mammary glands, prostate, and urinary bladder. MT4 can be found in stratified squamous epithelium. MT1 has 18 isoforms, including 10 functional genes: *MT1A*, *MT1B*, *Metallothionein 1E* (*MT1E*), *MT1F*, *MT1G1*, *MT1G2*, *MT1H*, *MT1HL1*, *MT1M*, and *MT1X* and 8 pseudogenes: *MT1CP*, *MT1DP*, *Metallothionein 1* pseudogene (*MT1JP*), *MT1L*, *MT1LP*, *MT1XP1*, *MT1P3*, and *MT1P1* [3–5]. As detailed in the following sections, metallothioneins play key roles in maintaining heavy metal balance, counteracting oxidative stress, regulating inflammatory processes, and modulating immune responses. Given that these processes are central to many liver pathologies, it can be expected that MT1 and MT2—along with their various isoforms—are likely to play a significant role in the development and progression of various liver diseases.

MTs have been found to play a regulatory role in maintaining heavy metal homeostasis in the cell; this is the maintenance of correct levels of the two main physiological metals, Zn and Cu [6], acting as a central storehouse for intracellular Zn and mediator of intracellular Zn signaling [7]. Later studies confirmed the protective effect of MTs not only against Cd toxicity [8, 9] but also in preventing toxicity from other heavy metals. This includes initiating the detoxification process in the presence of Cu, Zn, arsenic (As), and chromium (Cr) [3, 10, 11].

The expression of MTs is induced not only by metal ions (such as Cu, Zn, Cd, As, and Cr) but also by free oxygen and nitrogen species (ROS and NOS) and other free radicals; proinflammatory cytokines (IL-1, IL-6, TNF-alpha, and IFN-gamma); bacterial endotoxin-lipopolysaccharide (LPS); and glucocorticosteroids [3, 12, 13].

Following their discovery nearly 70 years ago, it has become increasingly apparent that metallothioneins exhibit complex pleiotropic functions: MTs have been shown to counteract oxidative stress [8, 9], play a protective role against carcinogenesis and mutagenesis caused by ionizing radiation and chemical agents [14], provide protection against brain injury, and present antiapoptotic effect [11]. A vast body of evidence has been collected indicating the immunological involvement of MTs in central nervous system diseases and cancer [4, 15–22]. In recent years, emerging evidence indicates a more expansive involvement of MTs in inflammatory conditions and adaptive immunity [3, 22–27].

Oxidative stress has been found to lie at the pathogenic foundation of many diseases, including cancer, neurodegeneration, inflammatory, and cardiovascular diseases [3, 25, 28–30]. There is substantial evidence describing the upregulation of MTs under oxidative stress, suggesting their protective role against reactive oxygen species [3, 11, 31–34]. MTs have been found capable of scavenging ROS, thereby protecting critical cellular components such as DNA, proteins, and lipid membranes from damage [34, 35]. In MTs-deprived animal models, higher sensitivity to oxidative stress was observed, along with an inferior response to inflammatory and infection processes [6].

This study seeks to provide a comprehensive overview of metallothioneins in various liver pathologies. Specifically, we will focus on conditions characterized by inflammatory and oxidative stress backgrounds while also addressing pertinent literature regarding hepatic tumors.

## 2. Role in Metabolic Steatotic Liver Disease Pathogenesis: Oxidative Stress and Obesity

Metabolic dysfunction–associated steatotic liver disease (MASLD), formerly known as nonalcoholic fatty liver disease (NAFLD) [36], is an escalating public health concern, with its incidence reaching 30% of the global population [37]. The spectrum of MASLD ranges from simple hepatic steatosis (MASL, metabolic dysfunction–associated steatosis of the liver) to metabolic dysfunction–associated steatohepatitis (MASH), culminating in MASLD-associated cirrhosis. Key risk factors for MASLD development include obesity, diabetes and prediabetic states, hypertension, and hyperlipidemia [38, 39].

MASLD is a multifactorial condition influenced by both genetic and environmental factors, which are still being actively researched. The most established pathogenetic mechanisms are insulin resistance, dysfunctional adiponectin regulation, oxidative stress, lipotoxicity, obesity, and intestinal microbiota dysbiosis [38].

According to the most established hypothesis, insulin resistance causes an increased influx of free fatty acids (FFAs) to the liver by inducing lipolysis in visceral fat tissue. Additionally, it inhibits a very low-density lipoprotein (VLDL) production and impairs the removal of triglycerides (TGs) from hepatocytes, leading to TG accumulation within these cells [38]. This accumulation triggers an inflammatory process, resulting in the disruption of mitochondria and the endoplasmic reticulum, along with the production of ROS. Consequently, oxidative stress, lipid peroxidation, and the release of inflammatory cytokines occur. This chain of events ultimately leads to inflammation and subsequent fibrosis within hepatocytes, resulting in MASH [40].

MASH is significantly influenced by oxidative stress, which is considered a key pathogenic factor in its' development [41]. However, contrary to the upregulation expected in the context of oxidative stress, MASLD-specific studies reveal a downregulated expression of metallothioneins. In the animal model of Type 2 diabetes with diet-induced liver steatosis, metallothionein 1 (MT1) and MT2 expression in the liver tissue was significantly lower compared to the control group. Given the critical role of MTs in modulating liver mitochondrial function, this downregulation is thought to contribute to the development of steatohepatitis and obesity [42].

### 2.1. Obesity

MTs appear to play a role in obesity, which is both a risk factor and a concomitant condition of MASLD. MT1 and MT2 knockout mice are moderately obese, suggesting the role of MTs in the regulation of energy balance [43]. In an animal study by Du et al. [44], mice fed with a high-glucose diet for 4 weeks presented insulin resistance at higher body weight, elevated FFA accumulation in the liver, along with downregulation of MT2 levels. In another murine study by Giller et al. [45], mice were exposed to dietary restriction (75% of normal diet for 6 months) followed by 6 months of ad libitum refeeding. This group was compared with an ad libitum–fed control group. MT1 was increased in the group with dietary restriction [45]. Considering the biological modulating activity of MTs on liver mitochondria, the downregulation of MTs was thought to contribute to the development of steatohepatitis and obesity [42].

Group studying mutations in the *MT1E* gene in a three-generation family with diabetes found that carriers of the *MT1E p.C36Y* mutation demonstrated increased weight gain, elevated postchallenge serum glucose, elevated liver enzyme levels, and hepatic steatosis. Conversely, mutation of MT*1E p.C36C* stabilized glucose metabolism and reduced body weight [46].

These observations suggest a link between MTs expression and body weight regulation, implying that individuals with low MTs levels may be more susceptible to obesity and the development of steatohepatitis. Moreover, distinct mutations in MTs genes, such as those identified in *MT1E*, could further contribute to metabolic disturbances and liver pathology. Given the emerging evidence that the upregulation of MTs exerts protective effects against both obesity and steatohepatitis, targeting MTs pathways represents a promising therapeutic strategy for both obesity and MASLD.

### 2.2. Progression From MASL to MASH

In the progression from MASL to MASH, the downregulation of MT1 family members has been identified as an important molecular change. Li et al. [47] reported that *MT1M* is the only downregulated hub gene in this transition, suggesting that decreased *MT1M* expression may contribute to the worsening of liver conditions from simple steatosis to steatohepatitis. Guan et al. [48] similarly showed that MT1B expression is reduced in MASH liver tissues, high-fat diet mouse models, and FFA-induced hepatocytes. Additional studies have shown that the overall MTs expression is significantly lower in MASH compared to MASL and healthy controls, supporting the idea that MTs may play a protective role against the oxidative stress–induced liver damage observed in MASH [49]. An older study by Alscher et al. [12], which assessed MTs expression using immunohistochemical (IHC) staining, found no difference between steatotic and normal liver, a result that may align with newer findings suggesting that MTs downregulation is more closely linked to the progression toward steatohepatitis, not the simple steatosis.

Building on these molecular insights, the work by Guan et al. [48] points to important therapeutic opportunities. Their findings show that upregulating MT1B reduced TG and cholesterol levels, decreased lipid droplet accumulation, and lowered inflammatory markers, while silencing MT1B leads to increased lipid accumulation, heightened inflammation, and enhanced fibrosis in vivo. These observations suggest that targeting MT1B—and potentially other MTs isoforms—could offer a novel strategy to counteract lipid dysregulation, inflammation, and fibrotic progression in metabolic dysfunction–associated liver diseases. As for other therapeutic avenues, You et al. [50] found that a ketogenic diet alleviates steatosis in MASLD mice, with MT2 upregulated as a key effector mediating reduced hepatic oxidative stress. Collectively, the emerging evidence positions MTs not only as biomarkers of disease progression but also as promising therapeutic targets in MASLD.

## 3. Role in Hepatotoxicity

The induction of MTs enhances the liver's resilience against toxic insults from environmental and pharmacological agents. Intracellular MTs binds heavy metals and reduces oxidative stress, thereby mitigating cellular damage. MTs synthesis significantly protects animals from the hepatotoxic effects of chemicals such as ethanol, carbon tetrachloride, paracetamol, and Cd [9].

Ethanol is a known hepatotoxic xenobiotic, leading to a spectrum of liver pathologies, from simple steatosis to steatohepatitis, chronic hepatitis, and alcohol-related liver cirrhosis [39]. Oxidative stress is a significant aspect of ethanol-induced hepatotoxicity. It contributes to the progression of liver damage by promoting inflammation and immune responses [51].

### 3.1. Protective Role of MTs in Alcohol-Related Liver Disease (ArLD) and Alcoholic Hepatitis

It has been shown that overexpression of *MT1* in murine models improves ArLD by reducing hepatic oxidative stress and inflammation. Histological analysis indicated significant improvements in lipid peroxidation (4-HNE staining) and ROS levels in both control and study groups after *MT1* overexpression [52]. Additionally, microarray analysis on liver tissues from mice subjected to chronic-plus-binge ethanol feeding revealed significant hepatic upregulation of *MT1* and *MT2*. Genetic deletion of these genes worsens ethanol-induced liver injury, as evidenced by increased serum ALT levels, neutrophil infiltration, and oxidative stress, suggesting a protective role of MTs [53]. Furthermore, MTs-transgenic mice with hepatic overexpression of MTs and elevated Zn levels demonstrated resistance to ethanol-induced liver injury, while MTs-knockout mice with reduced hepatic Zn were more susceptible to alcohol toxicity [54].

Interestingly, MTs expression in ArLD is nuanced, with distinct isoform behavior. Grasselli et al. [55] found increased oxidative stress in ArLD patients, alongside MT1A downregulation and MT1E upregulation compared to controls. Moreover, MT1A expression showed a positive correlation with hepatic iron levels. The observed divergence in MT1A and MT1E expression patterns may reflect the nuanced biology and functional specialization of MTs isoforms. Specifically, MT1E is generally characterized by low basal expression across most tissues but demonstrates strong inducibility under stress conditions, whereas MT1A is constitutively highly expressed and exhibits poor inducibility in response to oxidative stress [56]. These findings align with broader evidence suggesting that metallothioneins contribute to the cellular defense against oxidative stress and imply that insufficient baseline MTs expression may play a contributory role in the pathogenesis of ArLD.

Collectively, these findings suggest that enhancing MTs expression may represent a promising therapeutic strategy to counteract hepatic oxidative stress and inflammation in ArLD, ultimately limiting ethanol-induced liver injury by leveraging the antioxidant and cytoprotective properties of MTs.

### 3.2. Chronic Alcohol Abuse and Alcohol-Related Cirrhosis

Studies have shown that patients with chronic alcoholic abuse or alcoholic cirrhosis generally do not exhibit elevated levels of MTs. In fact, these patients typically have MTs levels similar to healthy controls [12]. Furthermore, research has indicated that in alcohol-related liver cirrhosis, plasma MTs concentrations are generally within the normal range [57]. A possible explanation for this observation might involve the loss of hepatocytes in alcohol-related liver cirrhosis or perhaps the exhaustion or impairment of Zn-induced pathways and antioxidant defenses as a consequence of prolonged chronic inflammation [58].

## 4. Role in Autoimmune and Cholestatic Diseases of the Liver

MTs have been widely recognized for their modulatory role in various autoimmune and inflammatory diseases. Increased MTs expression has been observed during the acute phases of conditions such as inflammatory bowel disease (IBD), rheumatoid arthritis, atopic dermatitis, Parkinson's disease, and multiple sclerosis, where in some cases, MTs is thought to exert protective or therapeutic effects [3, 16, 23, 24, 59–62]. Despite this, there is limited literature exploring the regulatory function of MTs in liver-specific autoimmune diseases, including autoimmune hepatitis (AIH), primary biliary cholangitis (PBC), and primary sclerosing cholangitis (PSC).

### 4.1. AIH

Alscher et al. [12] reported a significant increase in MTs levels in the livers of patients with autoimmune diseases compared to controls. This increase was attributed to the acute-phase induction of MTs by proinflammatory cytokines. Notably, MTs staining exhibited a consistent nuclear pattern in these autoimmune conditions. However, the study did not specify which autoimmune diseases were examined, leaving some uncertainty regarding the specific conditions involved. Although studies specifically investigating the role of MTs in AIH are limited, some research has used liver samples from AIH patients as controls in studies on Wilson's disease (WD). In both Wiethoff et al. [63] and Rowan et al. [64], AIH samples demonstrated some degree of MTs staining.

Further research is needed to understand the role of MTs in the development of AIH, especially given their known involvement in other autoimmune and immune-related diseases, where they can help improve symptoms and support remission [3, 15, 23, 27, 61, 65].

### 4.2. PBC and PSC

PBC and PSC are distinct liver diseases with differing etiologies, pathologies, and clinical manifestations. Despite these differences, both conditions share an underlying autoimmune component and ultimately culminate in a similar pathological outcome—progressive intrahepatic cholestasis. This cholestasis drives ongoing hepatocyte injury, leading to fibrosis and, in advanced stages, cirrhosis [39].

Cholestasis is well documented to be associated with elevated Cu concentrations [12, 57], and since one of the primary roles of MTs is to regulate intracellular Cu, their upregulation in cholestatic diseases is expected, as a compensatory mechanism to mitigate Cu-induced hepatotoxicity [12, 57, 66].

In a study on plasma MTs concentration in liver disorders, researchers found significantly elevated levels in PBC and PSC patients. In PBC, MTs concentration increased as the disease progressed, correlating with serum total bilirubin [57].

In a study by Alscher et al. [12], examining MTs expression in liver biopsies across various disease etiologies, MTs levels were significantly higher in cholestatic samples compared to both normal liver tissue and other liver disease etiologies. These findings were further corroborated in other studies, where PBC and PSC exhibited the second-highest intensity of MTs staining, following WD [63, 64, 66].

Alscher et al. [12] demonstrated that only the cholestatic specimens showed a significant correlation between MTs levels and the degree of histological damage. Furthermore, MTs expression was significantly increased in the cytoplasm rather than the nucleus, with a notable concentration in bile plugs. By contrast, in inflammatory conditions such as IBD and autoimmune diseases, MTs was predominantly elevated in the nucleus. These distinct patterns suggest that MTs could serve as a potential diagnostic marker for cholestatic diseases [12]. Comparative studies examining MTs expression and MTs cellular distribution between AIH, PBC, and PSC could provide valuable insights into the potential of MTs to aid histopathological differential diagnosis, particularly in the challenging context of overlap syndromes among these conditions.

#### 4.2.1. Gut–Liver Axis Involvement in PBC and PSC

Microbiome imbalance and disruption of the gut–liver axis have garnered increasing scientific attention in the context of cholestatic liver diseases. In PSC, the importance of the gut–liver axis is reflected in its close association with IBD. Gut dysbiosis and gut–liver axis disruption are thought to contribute to bile duct inflammation and fibrosis through immune-mediated mechanisms [67–69]. In PBC, altered gut microbiota can induce PBC-like liver lesions and exacerbate liver injury, suggesting a causal role for dysbiosis in disease progression and immune activation [70, 71].

Building on these insights, another potential therapeutic strategy involving MTs can be identified in the context of liver diseases. A murine study investigating Cd-induced liver steatosis, inflammation, and fibrosis demonstrated that probiotic delivery of MTs, using engineered *Escherichia coli*, effectively mitigated the deleterious effects of Cd in the liver tissue and restored the integrity of the colonic epithelial barrier compromised by Cd exposure. Notably, MTs delivery increased beneficial gut bacteria (e.g., Verrucomicrobiota), helping to correct dysbiosis associated with liver disease and further supporting gut barrier function [72].

These findings raise compelling questions about the potential application of MTs-based microbiome-targeted therapies in cholestatic liver diseases, where gut–liver axis disruption plays a pivotal role in driving hepatic injury and immune-mediated pathology.

## 5. Role in WD

WD is a hereditary disorder impacting Cu metabolism due to mutations in the *ATP7B* gene, which normally encodes a Cu-transporting protein. This genetic defect results in toxic Cu buildup in the body, leading to a range of hepatic, neurological, and psychiatric symptoms [63]. Since the excessive Cu accumulation lay at the foundation of WD pathogenesis, increased expression of MTs is expected and was described from the 1980s [12, 66, 73]. The production of MTs is mainly controlled by metal-regulatory transcription factor-1 (MTF-1). When intracellular Cu levels rise, MTF-1 becomes activated and moves to the nucleus, where it binds to metal response elements (MREs) in the promoters of MTs genes, stimulating their transcription [63, 74, 75]. The upregulation of MTs is documented to exert a protective effect on hepatocytes in WD by sequestering free Cu, thus limiting its cytotoxic potential [76, 77]. Additionally, MTs plays an important antioxidant role, as their high cysteine content enables them to neutralize reactive oxygen species generated by Cu overload, thereby further mitigating oxidative stress and cellular damage [78].

Diagnosis of WD can be challenging due to the rarity of the condition, its multiorgan and nonspecific presentation, and the wide range of histologic appearances, with early WD often mimicking MASLD or other chronic liver diseases [63].

The classical histopathological staining used for detecting Cu deposits is rhodanine or orcein [64]. Rhodanine stain positivity rates in WD patients have been reported as approximately 60% [79]. Studies indicate a very good sensitivity, specificity, and accuracy of MTs IHC staining in WD diagnosis—85.7%, 96.9%, and 94.9%, respectively [63, 64]. MTs had higher sensitivity than rhodanine for the diagnosis of WD [64], specifically in the early stages of WD [63]. A possible explanation for this phenomenon lies within the fact that the Cu bound with MTs in the hepatocytes is in the reduced cuprous (Cu+) form, whereas detection by histochemical stains like rhodanine requires the oxidized cupric (Cu2+) form.

Multiple studies have shown that liver biopsies from patients with WD stained immunohistochemically for MTs showed an intense and specific staining pattern (moderate to strong staining in > 50% of hepatocytes) compared to the control group: steatohepatitis, chronic hepatitis, AIH, biliary diseases, and healthy livers [64, 80]. Even in cholestatic diseases like PBC and PSC, where MTs is also present, MTs staining helps in diagnosis because WD shows a distinct pattern—strong, widespread, perivenular staining in hepatocytes—which is less intense and differently distributed in PBC and PSC [63].

All cited studies suggest that MTs IHC staining could be widely used for the histopathological diagnosis of suspected WD cases. However, challenges such as the availability and standardization of MTs antibodies, the need for specialized IHC expertise, and economic considerations remain significant barriers to its broader adoption.

## 6. Role in Hepatic Malignancies

MTs have been extensively studied for their involvement in malignancy, particularly their roles in carcinogenesis and tumor progression, which underscores the potential implications of these proteins in both processes [81, 82]. Various MTs isoforms have been implicated in key aspects of carcinogenesis, including apoptosis, cell proliferation, and the sensitivity of cancer cells to different anticancer treatments [82]. Studies using animal models have suggested that MT1 and MT2 play a protective role not only against metal toxicity and oxidative stress but also against chemical and radiation-induced carcinogenesis [14]. In this context, we explore the role of MTs in hepatic malignancies, specifically hepatocellular carcinoma (HCC) and cholangiocarcinoma (CCC).

### 6.1. HCC

There is substantial evidence indicating that MT1 and MT2 expression in hepatocytes is downregulated in HCC cells. This reduction in expression, observed using IHC methods, contrasts with the surrounding normal hepatocytes [83–88] and further confirmed in molecular studies using to determine expression of the genes—the outcomes demonstrated the downregulation of MT1, MT2, and MTF-1 in human HCC as compared with normal tissues [89–92]. Moreover, Fu et al. [84] have presented in the murine model that overexpression of MT1M inhibited cell viability, colony formation, cell migration, invasion, and cell apoptosis in HCC cell lines. Dong et al. [93] have also observed MT1M downregulation in HCC tumor tissue and proposed its role in oncogenesis initiation and progression of HCC.

Some authors propose a role of MTs as a potential biomarker for HCC diagnosis. In immunostaining, the sensitivity and specificity of MT1M for HCC diagnosis were 76.27% and 89.83%, respectively. Li et al. [89] have classified MT1 and MT2 as biomarkers for HCC, as their expression was downregulated in HCC and peri-HCC tissue. [94] have described isoforms MT1M and MT1G as potential noninvasive biomarkers for HCC, as their serum concentration was significantly higher than in patients with chronic hepatitis B and was positively correlated with the tumor size. MTs genes (including MT1E, 1F, 1G, 1H, 1J, 1M, and 1X) have also been described as biomarkers to predict recurrence of HCC [91].

#### 6.1.1. MTs and HCC Prognosis

There is a substantial body of research revolving around the involvement of MTs in HCC progression and overall prognosis. The loss of nuclear expression of MT1 and MT2 is correlated with high Edmondson–Steiner grade of tumor and the presence of microvascular invasion and may predict prognosis in patients with HCC [87]. *MT3* has been described as one of seven identified genes, whose expression was inversely correlated with HCC survival time [95]. MT1M expression was associated with poor HCC prognosis in patients who underwent a curative resection [96]. MT1G and MT1H were downregulated in HCC patients; along with high serum Cu concentration, this was related to reduced survival in HCC [97].

#### 6.1.2. Treatment Opportunities

The role of MTs has also been studied in the context of therapeutic options.

MTs levels increased after the administration of lenvatinib [98]. MT1G has been found to be a critical regulator and promising therapeutic target of sorafenib resistance in human HCC [62]. Similarly, MT3 has been found to be involved in processes driving resistance to sorafenib; hence, it emerged as a potential druggable therapeutic target [99]. A study by Liu et al. presented that MT1E suppressed HCC cell growth in vitro and in vivo and proposed that MT1E could induce apoptosis and suppress the metastasis of HCC cells [90].

### 6.2. CCC

The role of metallothioneins in CCC has been studied way less extensively than in HCC. However, genes from the metallothionein family (viz., *MT1A*, *MT1E*, *MT1F*, *MT1G*, *MT1H*, *MT1X*, and pseudogene *MT1-IP*) were significantly downregulated in liver samples from patients with CCC [100]. In IHC staining of both intra- and extrahepatic CCC, strong metallothionein expression is identified as an independent poor prognostic parameter [101]. According to a study by Zhao et al., *MT1JP* alleviated proliferation, migration, and invasion and induced apoptosis in CCC cells, thus proving to be a diagnostic or therapeutic target in CCC [102].

## 7. Role in Viral Hepatitis

Metallothioneins play an important role in the body's response to infections and have been reported to be upregulated in various infectious conditions, both viral and bacterial [3, 103–107]. The proposed mechanisms underlying this upregulation involve the role of MTs in mitigating mitochondrial stress and regulating autophagy, processes that are activated during viral infections [108]. Additionally, MTs mitigates oxidative stress caused by immune cells and bacteria themselves [109].

### 7.1. HCV Infection

MTs has been shown to be upregulated in in vitro studies of HCV infection [108, 110]. This response is thought to be driven by increased intracellular Zn levels, which may contribute to antiviral defense by inhibiting viral protein translation and supporting host antiviral signaling pathways [111]. Immunofluorescence studies have shown MTs accumulation in the nuclei of HCV-infected hepatocytes, suggesting a role in modulating transcriptional responses—potentially through the regulation of genes linked to apoptosis and cell survival [108]. Additionally, MTs protects against oxidative stress by neutralizing reactive oxygen species, reducing hepatocyte injury and potentially slowing liver disease progression [112].

However, comparable upregulation has not been reported under in vivo conditions. Researchers attributed this to the chronic nature of in vivo infections, suggesting that MTs upregulation is more closely associated with acute infection, as seen in in vitro models [108]. Similar findings have been confirmed in other in vivo studies, where no increase in MTs expression was detected compared to controls [12, 57], and in some cases, MTs expression was found to be downregulated [113–115]. Only one in vivo study reported MTs upregulation in hepatocytes from patients with chronic HCV infection [116].

Some studies [113, 116] demonstrated that MTs expression in HCV-infected livers inversely correlated with the inflammatory activity of the disease and the stage of fibrosis in hepatocytes. In these cases, observed downregulation was associated with low IL-6 levels and Zn deficiency [113].

While in vitro studies suggest that MTs may be upregulated during acute infection, in vivo studies indicate that chronic HCV infection is often accompanied by downregulation of MTs expression. This downregulation may reflect more advanced disease stages, including fibrosis. These findings seem to be in keeping with the postulated protective role of MTs against HCV infection, potentially due to their mild antiviral activity, which is linked to the modulation of intracellular Zn homeostasis [108, 113]. Interventions that reduce MTs expression via RNA interference in cultured cells have resulted in enhanced viral replication, affirming the role of MTs in modulating antiviral defense mechanisms [108].

Summarizing, lower MTs expression may reflect the chronic nature of liver inflammation in HCV, signaling more advanced disease stages. This connection is particularly compelling in the context of HCC surveillance, as prolonged infection and sustained inflammation increase the risk of malignant transformation [117], making MTs levels a potentially valuable biomarker for identifying patients at heightened risk of HCC.

### 7.2. HBV Infection

The involvement of MTs in HBV pathogenesis and progression to cirrhosis or regeneration remains underexplored, though some studies have investigated this link. For instance, interactions between HBV antigens (HBeAg, HBcAg, and HBxAg) and MTs have been demonstrated in vitro, suggesting it may participate in the pathogenesis of hepatitis B through direct molecular interactions with viral components [118]. In another study, hepatocytes infected in vitro with HBV showed only slight MTs upregulation, much weaker than the MTs increase observed after HCV infection or Zn treatment [111].

The natural course of HBV infection involves chronic liver injury and inflammation, followed by hepatocellular regeneration. These processes expose dividing cells to elevated levels of ROS, which are believed to cause DNA damage and, ultimately, neoplasia. Given the radical-scavenging properties of MTs, researchers utilized an HBsAg-positive animal model with 10-fold overexpression of MT1 to examine its effect on HBV-induced pathology. Their findings showed that MT1 mitigated HBV-induced hepatitis, with a reduction in hyperplasia and aneuploidy. However, the expression of MT1 did not fully prevent hepatitis or neoplasia [119].

In human livers chronically infected with HBV, MT1 and MT2 have been found to be downregulated compared to healthy liver tissue. This downregulation has been attributed to the chronic nature of the infection, and its inverse correlation with biochemical markers of liver injury suggests a protective role of MTs expression in the liver undergoing chronic inflammatory processes [115].

These findings suggest a potential protective role of MTs in HBV infection, possibly through combating ROS; however, the evidence gathered so far is less robust than that in other discussed diseases and requires further research to explore therapeutic opportunities.

## 8. Summary

Metallothioneins are primarily recognized for their roles in cellular metal homeostasis and protection against metal toxicity. However, their function in mitigating oxidative stress and modulating immune responses has emerged in the pathogenesis of various liver diseases. Studies suggest a link between MTs expression and body weight, indicating that individuals with low MTs levels may be more susceptible to obesity and steatohepatitis. Furthermore, the downregulation of MTs is thought to contribute to disease progression from simple steatosis to steatohepatitis.

In ArLD, MT1 overexpression appears protective, reducing oxidative stress and inflammation in alcoholic hepatitis. However, in advanced stages of alcohol-related cirrhosis, MTs levels remain within normal ranges, possibly due to hepatocytes loss.

In PBC and PSC, MTs exhibits intense expression with distinctive cellular patterns, correlating with disease progression, making them promising diagnostic markers. In WD, MTs serves as a valuable diagnostic tool, offering excellent sensitivity and specificity compared to rhodanine staining. While AIH studies confirm MTs expression, its significance remains unclear, warranting further exploration given MT's established role in other autoimmune diseases.

In HCC, MTs downregulation is linked to poor prognosis and reduced survival. Independently, MTs has been postulated as a biomarker with good sensitivity and specificity and has attracted attention as a therapeutic target, particularly in overcoming sorafenib resistance. In viral hepatitis (HBV/HCV), MTs expression is upregulated in vitro but downregulated in vivo, reflecting chronic inflammation and fibrosis. MTs may thus play a protective role in these conditions.

Future research should aim to further elucidate the precise mechanisms by which MTs contribute to liver disease progression and explore their potential as therapeutic targets, particularly in conditions where MTs modulation could alter disease outcomes and improve patient prognosis.

## Data Availability

Data sharing is not applicable to this article as no datasets were generated or analyzed during the current study.
